# High-risk patients benefit most from nifedipine GITS–telmisartan combination

**Published:** 2010-08

**Authors:** J Aalbers

**Affiliations:** Special Assignments Editor

## Introduction

Two very effective antihypertensive medications with well-established and significant cardiovascular outcome studies have been combined and used for the first time in early combination therapy in the TALENT study. Results from this multicentre, prospective, randomised, doubleblind trial were announced at the 2010 European Society of Hypertension (ESH) congress held in June in Oslo, Norway, and highlighted the rapid, safe and effective blood pressure-lowering action of this combination.^1^

The TALENT (Study evaluating Efficacy of Nifedipine GITS – Telmisartan combination in Blood Pressure Control and Beyond: Comparison of Two studies) enrolled 405 patients with office systolic blood pressure at a baseline of ≥ 135 mmHg and with a high cardiovascular risk because of diabetes, the metabolic syndrome, and echocardiographic/ECG evidence of left ventricular hypertrophy or microalbuminuria. Patients could be admitted to the trial if other antihypertensive medication (ACE inhibitors, other ARBs, or CCBs) could be safely withdrawn.

**Fig. 1.**
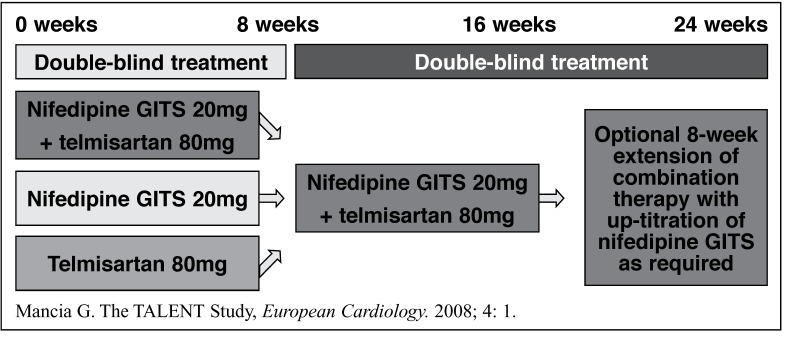
TAL ENT study design.

Patients were randomised to initial administration of telmisartan (80 mg/day) plus nifedipine GITS (20 mg/d), telmisartan alone, or nifedipine GITS alone in a 2:1:1 ratio. Treatment was continued for 24 weeks, shifting the monotherapy groups to combination therapy after eight weeks (Fig. 1).

Office and ambulatory blood pressure was measured after two, eight, 16 and 24 weeks and after eight, 16, and 24 weeks, respectively. Up-titration occurred when needed but not to blood pressure levels below 120 mmHg.

## Results

Initiating treatment with the combination therapy resulted in earlier blood pressure control. This was maintained throughout the study period both with regard to office and ambulatory blood pressure control, which was reduced by 14.2/3 mmHg and 10/4.7 mmHg, respectively.

Both combination and monotherapy substantially lowered systolic and diastolic blood pressure. The 24-hour data showed that the effect was consistent throughout the 24-hour period. Of importance is that longer-term control was similar, irrespective of the initial monotherapy treatment strategy followed or whether the combination was initiated first.

In terms of the evidence-based reduction of cardiovascular outcomes, telmisartan in the ONTARGET^2^ studies and nifedipine GITS in the ACTION,^3^ INSIGHT,^4^ and ENCORE5 trials have best-in-class results. This evidence, together with the South African and international guidelines’ emphasis on the use of early effective antihypertensive agents in high-risk patients, raises the importance of the TALENT results in everyday clinical practice.

## References

[1] Mancia G, Parati G, Parati G, Parati G, Bilo G, Ruilope L (2010). Early blood pressure control by the nifedipine GITS/telmisartan combination.. Abstract..

[2] (2008). Telimsartan, ramipril, or both in patients at high risk of vascular events.. N Engl J Med.

[3] Poole-Wilson PA, Lubsen J, Kirwan BA (2004). Effect of long-acting nifedipine on mortality and cardiovascular morbidity in patients with stable angina requiring treatment (ACTION trial): randomised controlled trial.. Lancet.

[4] Brown MJ, Palmer CR, Castaigne A (2000). Morbidity and mortality in patients randomised to double-blind treatment with a long-acting calcium-channel blocker or diuretic in the International Nifedipine GITS study: Intervention as a Goal in Hypertension Treatment (INSIGHT).. Lancet.

[5] (2003). Effect of nifedipine and cerivastatin on coronary endothelial function in patients with coronary artery disease: the ENCORE I study (Evaluation of Nifedipine and Cerivastatin On Recovery of coronary Endothelial function).. Circulation.

